# Early-life physical activity and risk of juvenile idiopathic arthritis: a longitudinal ABIS cohort study using a symptom-free baseline design

**DOI:** 10.1186/s12969-026-01266-9

**Published:** 2026-09-01

**Authors:** Noman Sohail, Erik Kindgren, Johnny Ludvigsson

**Affiliations:** 1 Division of Pediatrics, Department of Biomedical and Clinical Sciences, Linköping University, Linköping, SE 58185 Sweden; 2 Center for Social and Affective Neuroscience, Department of Biomedicial and Clinical Sciences, Linköping Univeristy, SE 58185 Linköping, Sweden; 3 Skaraborg Institute of Research and Development, Skövde, Sweden; 4 Gillberg Neuropsychiatry Centre, University of Gothenburg, Gothenburg, Sweden; 5 Crown Princess Victoria Children´s Hospital, Region Östergötland, Linköping, SE 58185 Sweden

**Keywords:** Physical activity, Juvenile idiopathic arthritis, Joint pain, Longitudinal cohort, ABIS

## Abstract

**Background:**

The role of physical activity (PA) in the development of juvenile idiopathic arthritis (JIA) remains unclear, and the evidence addressing this question is limited. Therefore, this study aimed to investigate the longitudinal association between early-life PA and the risk of JIA, using a symptom-free baseline design to minimize bias from early disease manifestations.

**Methods:**

A total of 16,415 participants were included from the All Babies in Southeast Sweden cohort. PA was assessed at age 5, 8, and 10–12 years using a composite Total Activity Score by categorizing participants into less active and highly active groups based on z-score threshold. Incident JIA cases (*n* = 88) were identified through Swedish National Patient Registers. At each age, individuals with a diagnosis of JIA prior to or at the time point were excluded. Participants reporting joint symptoms (pain, swelling, limited mobility, or limping) were excluded to create a symptom-free baseline population. Remaining participants were followed for incident JIA. Cox proportional hazard models were used to estimate hazard ratios (HRs) with 95% confidence interval. All models were adjusted for sex, parental education, and family history of rheumatic disease.

**Results:**

After exclusions, 6832 participants were identified at age 5, 3885 at age 8, and 4445 at age 10–12. A total of 62, 56, and 40 incident JIA cases during follow-up were identified in the respective age-specific cohorts. Higher PA was consistently associated with a reduced risk of JIA across all age groups, with HRs ranging from 0.72 to 0.88 in unadjusted models, and 0.76 to 0.91 in adjusted models. However, none of the associations reached statistical significance.

**Conclusion:**

Our findings suggest that PA in childhood does not increase the risk of JIA although any potential protective association is likely modest. The use of a symptom-free cohort strengthens causal interpretation by minimizing reverse causation.

**Graphical Abstract:**

**Figure:**
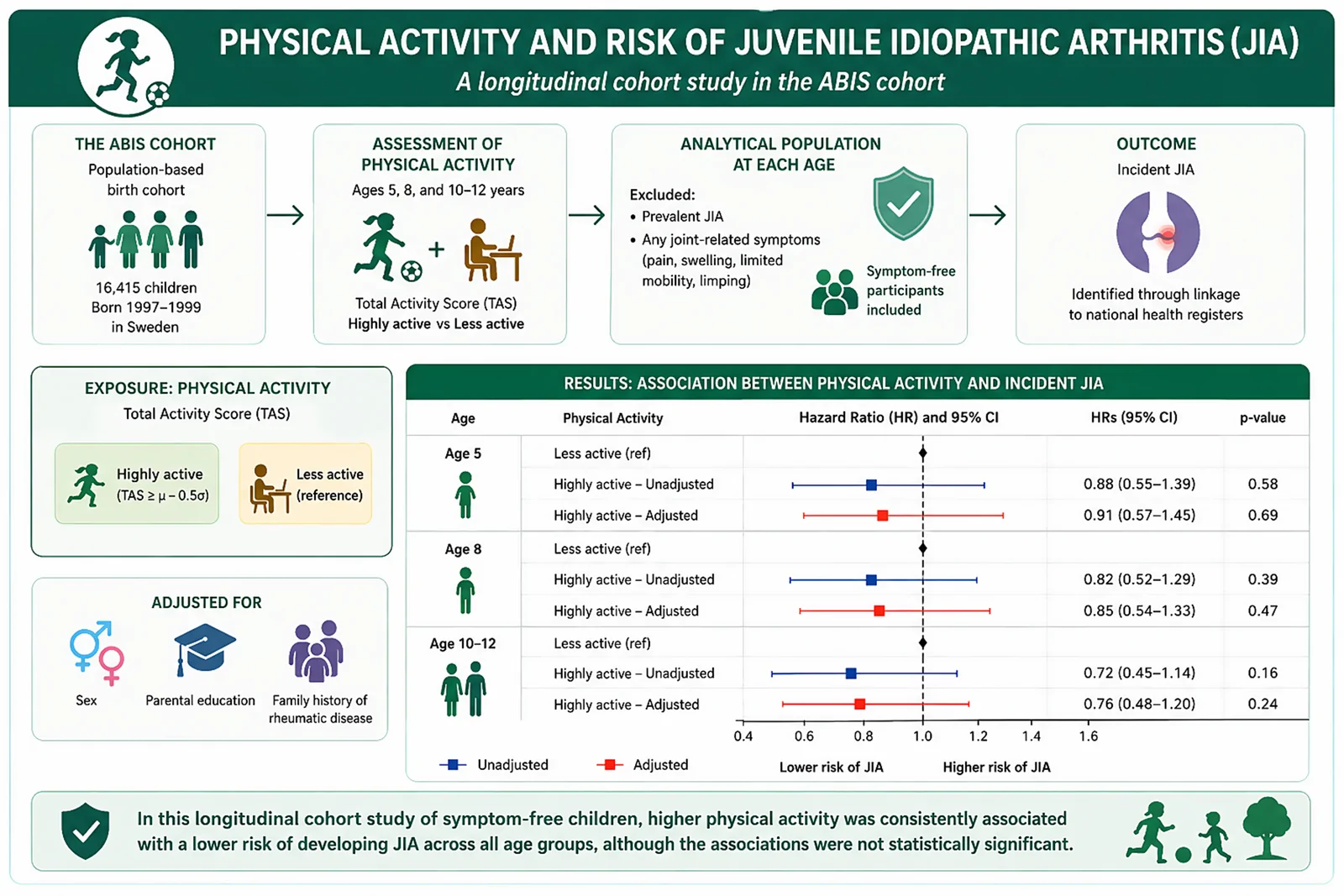

**Supplementary Information:**

The online version contains supplementary material available at https://doi.org/10.1186/s12969-026-01266-9.

## Why was this study needed?

• To investigate whether higher PA in early childhood is associated with the risk of developing JIA, while accounting for potential bias from early joint symptoms.

**Supplementary Information:**

The online version contains supplementary material available at https://doi.org/10.1186/s12969-026-01266-9.

## What it found?

• In a large prospective birth cohort, higher PA was associated with a consistent inverse trend in JIA risk across multiple age groups; however, these associations were not statistically significant when analyses were restricted to symptom-free individuals.

**Supplementary Information:**

The online version contains supplementary material available at https://doi.org/10.1186/s12969-026-01266-9.

## The implications for clinical practice?

• Our findings suggest that PA is unlikely to have a strong independent effect on JIA risk but may play a modest role. The study highlights the importance of accounting for early disease manifestations in epidemiological research. Further large-scale studies might needed to clarify the role of PA in the development of autoimmune diseases in children.

**Supplementary Information:**

The online version contains supplementary material available at https://doi.org/10.1186/s12969-026-01266-9.

## Background

Juvenile idiopathic arthritis (JIA) is the most common chronic rheumatic disease of childhood and is associated with pain, functional limitation, and reduced quality of life [1, 2]. In children with established JIA, Physical Activity (PA) and structured exercise are generally considered safe and have been associated with improvements in physical function and fitness [3–5].

In contrast, evidence regarding PA as a risk factor before disease onset remains limited. Most existing pediatric studies have focused on disease management after diagnosis rather than on etiological factors preceding JIA development. This represents an important gapin understanding potentially modifiable early-life exposures [6, 7].

The etiology of JIA is complex and multifactorial. Beside genetic factors, environmental and lifestyle-related exposures are increasingly recognized as potential contributors to immune dysregulation and disease development. Previous research has primarily focused on infections, microbiome composition, and perinatal factors [8–12]. However, PA is a biologically plausible exposure, as it influences immune regulation, systemic inflammation, and metabolic pathways [13, 14].

Evidence from adult populations suggests that PA may play a protective role in autoimmune diseases. For example, higher levels of leisure-time PA have been associated with a reduced risk of Rheumatoid Arthritis (RA) and other inflammatory conditions in prospective cohort studies [15–17]. Recent studies have also demonstrated that regular PA is associated with lower systemic inflammation and improved immune function, supporting its potential role in modulating autoimmune disease risk [13, 18]. However, direct extrapolation of these findings to pediatric populations should be made with caution, as disease mechanisms, exposure timing, and behavioral patterns differ substantially between children and adults.

A major methodological challenge in studying PA and JIA risk is the issue of reverse causation. Early, subclinical manifestations of JIA such as joint pain, stiffness, or reduced mobility, may occur years before formal diagnosis and can lead to decreased PA levels. This may create a misleading association in which low PA appears to increase disease risk, whereas in reality early disease symptoms may be responsible for reduced activity. Therefore, careful study design is required to ensure that PA precedes disease onset.

The All Babies in Southeast Sweden (ABIS) cohort provides a unique opportunity to address this question. The cohort includes repeated longitudinal assessments of PA and joint-related symptoms during childhood, combined with register-based follow-up for JIA diagnosis. This allows for the construction of a symptom-free baseline population at each age, minimizing the influence of early disease manifestations and strengthening causal interpretation.

In this study, we aimed to investigate the longitudinal association between PA at ages 5, 8, and 10–12 years and the subsequent risk of JIA, using a symptom-free baseline design to reduce bias from reverse causation.

## Methods

### The study population

This study was conducted within the ABIS cohort, a large population-based prospective longitudinal study designed to investigate genetic and environmental determinants of immune-mediated diseases [19]. The cohort initially invited 21,700 mothers who gave birth between October 1997 and October 1999, of whom 17,055 (78.6%) agreed to participate and provided informed consent. After excluding individuals with incomplete baseline data, 16,415 (75.7%) participants were available for analysis.

**Fig. 1:**
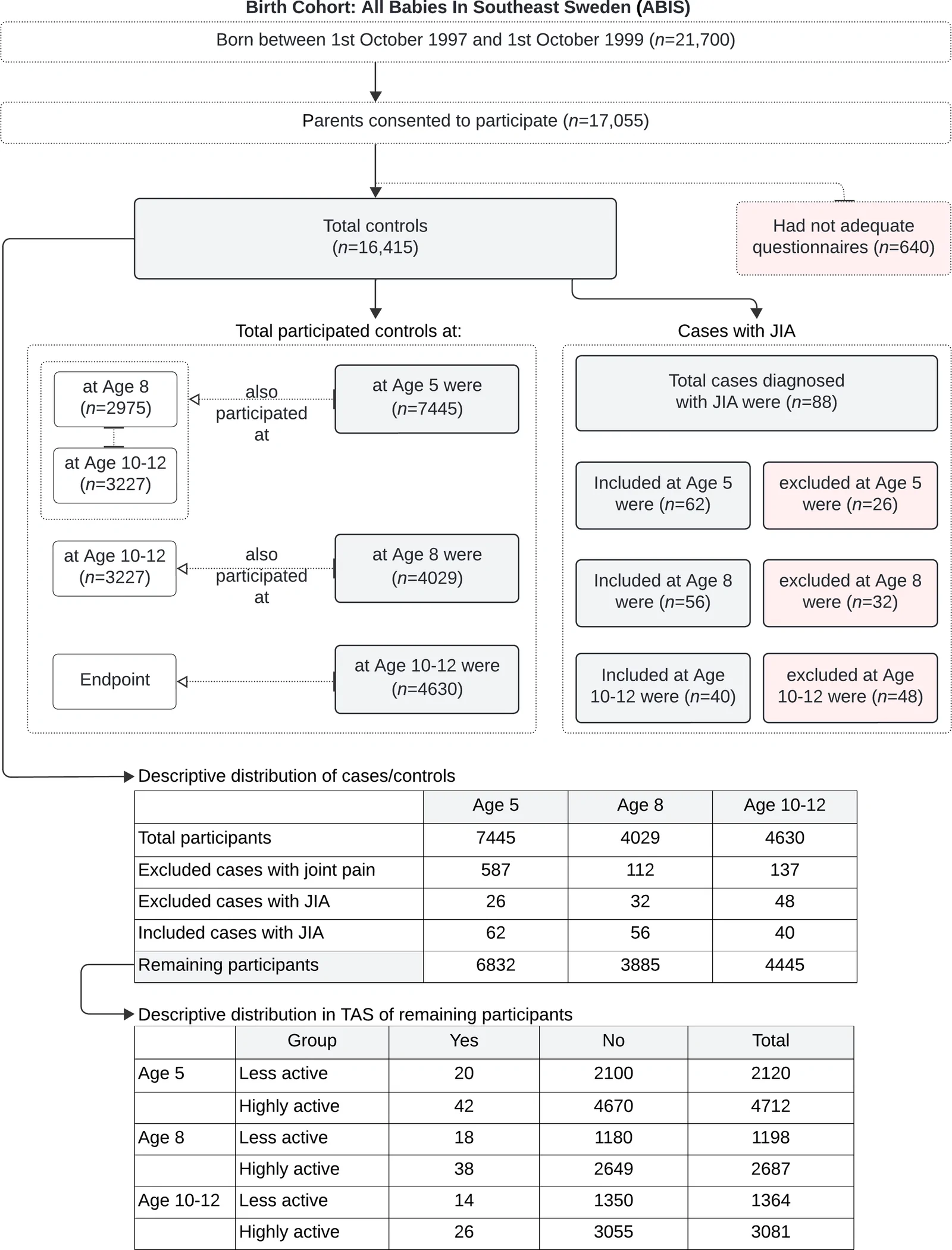
Flow diagram of the ABIS study population and follow-up

For the present study, data were analyzed at ages 5, 8, and 10–12 years (Fig. 1). Follow-up assessments were conducted longitudinally, with additional follow-up into young adulthood through linkage to national health registers.

### The ABIS cohort

The ABIS study is a population-based prospective birth cohort designed to investigate environmental and genetic determinants of immune-mediated diseases. Data collection was conducted through repeated questionnaires completed by parents during early childhood and by both parents and children at later follow-ups, including ages 5, 8, and 10–12 years.

The questionnaires covered a wide range of factors, including PA, lifestyle behaviors, and health outcomes. In addition, linkage to national health registers enabled long-term follow-up and objective ascertainment of clinical diagnoses, including JA. The longitudinal design, repeated exposure assessments, and high-quality register linkage provide a robust framework for studying early-life risk factors for disease development. More details about ABIS can be found on ABIS website (www.abis-studien.se).

### Outcome definition: juvenile idiopathic arthritis

The primary outcome was incident JIA, identified through linkage to the Swedish National Patient Register, which was launched in 1964, and is maintained by the Swedish National Board of Health and Welfare. A population-based register captures over 99% of all psychiatric and somatic hospital discharges, along with outpatient visits from both public and private healthcare providers. The register contains several types of information, including International Classification of Diseases (ICD) diagnostic codes and the personal identity number (PIN), which is a unique 10-digit identifier assigned to every resident in Sweden [20].

JIA cases were defined using ICD codes, including M08.0 (juvenile rheumatoid arthritis, seropositive), M08.1 (seronegative), M08.2 (systemic onset), M08.3 (oligoarticular), M08.4 (polyarticular), M08.9 (unspecified), and M09 (juvenile arthritis in diseases classified elsewhere).

The date of first recorded diagnosis was used to define the event time. Diagnoses recorded after the age of 16 years were retained only if they were considered to reflect JIA with onset before age 16. A total of 88 individuals were diagnosed with JIA during the follow-up period. To ensure appropriate temporal ordering between exposure and outcome, individuals diagnosed with JIA prior to or at the respective age of PA assessment were excluded from each age-specific analysis.

### Physical activity classification

PA was assessed at each ages 5, 8, and 10–12 years using a composite Total Activity Score (TAS), derived from questionnaire-based variables capturing both active and sedentary behaviors (see Additional file 1, Table S1). The TAS components were selected to reflect multiple dimensions of habitual PA, including structured exercise, unstructured movement, and sedentary behaviors, based on available ABIS questionnaire items.

To ensure the comparability across variables with different scales, all variables were normalized to a common range (0–1) using the standardized normalization procedures [21, 22]. Sedentary variables were reverse-coded prior to normalization so that higher values consistently represented higher levels of PA. All variables contributed equally to the TAS to avoid introducing subjective weighting and to ensure transparency and reproducibility of the composite score.

However, variables measured in hours per week were normalized by dividing the actual value by the maximum possible value of 168 h (representing a full week). Variables with hours per day were normalized by dividing by 24 h, and variables with frequency per week were normalized by dividing by seven, corresponding to the number of days in a week.

The final TAS was calculated as the mean of all normalized variables. Participants were categorized into two groups based on a z-score-derived threshold: less active (TAS < µ − 0.5σ) and highly active (TAS ≥ µ − 0.5σ) [23, 24]. This threshold was selected to maintain stable group proportions across age groups and to minimize misclassification bias. Although the TAS is not a previously validated instrument, internal consistency was high (Cronbach’s α ≈ 0.98), supporting the reliability of the composite measure.

Moreover, we selected this threshold (µ − 0.5σ) based on its grounding in standard deviation theory under the assumption of normality. In a standard normal distribution, approximately 31% of values fall below this threshold, making it a statistically interpretable and non-arbitrary point of division. This avoids the subjectivity associated with percentile-based cutoffs and ensures comparability across age groups. Further, applying stricter thresholds (µ − 0.75σ or µ − 1σ) led to a lower proportion of participants being classified as less active and the relative proportions also not stable across age groups (see Additional file 1, Table S2). The use of (µ − 0.5σ), resulting in a consistent ~ 31% classification rate, therefore provides both theoretical justification, empirical stability, and strengthening the methodology of the analysis.

### Joint symptoms assessment

Joint-related symptoms were assessed at age 5, 8, and 10–12 years using questionnaire data. The assessment included four questions addressing different aspects of musculoskeletal symptoms including joint pain, joint swelling, limited joint mobility, and limping.

Each variable was treated as a binary variable (yes/no). A composite variable (“joint pain”) was defined such that participants reporting at least one symptom were classified as having joint-related symptoms.

This composite approach allowed for comprehensive identification of early musculoskeletal symptoms, including both pain and functional impairment, which may represent early manifestations of rheumatic disease.

### Analytical framework

Age-specific prospective analyses were conducted at ages 5, 8, and 10–12 years. At each age, a symptom-free baseline population was defined to ensure that PA preceded any detectable disease process. This was achieved through (1) the exclusion of prevalent JIA cases, where individuals diagnosed with JIA prior to or at the respective age were excluded; (2) the exclusion of participants with joint symptoms, where individuals reporting any joint-related symptoms at the respective age were excluded.

This approach minimizes potential bias due to reverse causation, whereby early disease symptoms may influence PA levels prior to diagnosis. The remaining participants were followed for incident JIA occurring after the respective age.

### Covariates and confounders

The adjusted analyses included sex, parental education, and family history of rheumatic disease. These variables were selected a priori based on biological plausibility and their established associations with both PA and JIA risk [25].

Although additional covariates such as body mass index, dietary patterns, and socioeconomic indicators were available within the cohort, but were not included in the primary models to avoid overadjustment and model instability given the relatively small number of outcome events.

### Statistical analysis

Cox proportional hazards regression models were used to estimate hazard ratios (HRs) and 95% confidence intervals (CIs) for the association between PA and incident JIA. The age-specific analyses were not statistically independent, because the same participant could contribute to more than one analytical sample if eligible at multiple follow-ups. Therefore, estimates from the three age-specific models were interpreted as complementary analyses at different developmental stages rather than as independent replications.

Less active participants were used as the reference group. Both unadjusted and adjusted models were fitted. Adjusted models including sex, parental education, and family history of rheumatic disease. Missing data for PA variables were minimal (< 5%) and handled using available-case averaging within the TAS calculation [22], provided that sufficient items were present. All analyses were conducted using IBM SPSS Statistics (version 29.0) and Python (version 3.12) with standard scientific computing libraries. A two-sided p-value < 0.05 was considered statistically significant.

## Results

Table 1 shows the distribution of participants across different age groups, showing a slightly higher proportion of males in each group. A total of 7445, 4029, and 4630 participants had available data at ages 5, 8, and 10–12 years, respectively. Across all age groups, approximately 31% of participants were classified as less active and 69% as highly active based on the TAS threshold classification. The results of Cronbach’s alpha indicated excellent internal consistency for the TAS variables across all age groups (α = 0.98) (see Additional file 1, Table S3).

**Table 1:** Descriptive analysis of ABIS study samples

		Participants/Cases	Male	Female
Total ABIS	-	16,415	8485	7930
Participated at	Age 5	7445	3850	3595
	Age 8	4029	2100	1929
	Age 10–12	4630	2391	2239
Total Activity Score (TAS)				
Less active	Age 5	2308	1178	1130
	Age 8	1249	599	650
	Age 10–12	1435	761	674
Highly active	Age 5	5137	2672	2465
	Age 8	2780	1501	1279
	Age 10–12	3195	1630	1565
Diagnosed cases with JIA in cohort	-	88	29	59
Diagnosed cases with JIA at	Age 1	2	1	1
	Age 2	8	3	5
	Age 3	7	3	4
	Age 4	7	2	5
	Age 5	2	0	2
	Age 6	3	2	1
	Age 7	2	0	2
	Age 8	1	1	0
	Age 9	2	0	2
	Age 10	6	2	4
	Age 11	6	3	3
	Age 12	2	2	0
	Age 13	6	3	3
	Age 14	5	2	3
	Age 15	4	0	4
	Age 16	18	5	13
	Age 17	7	0	7
	Age 18	0	0	0
Developed joint pain	before Age 5	587	353	234
	from Age 5 to Age 8	112	67	45
	from Age 8 to Age 10–12	137	74	63

During follow-up, a total of 62, 56, and 40 incident JIA cases were identified from the respective cohort (Fig. 1). After excluding individuals diagnosed with JIA prior to or at each age, 7419 participants at age 5, 3997 at age 8, and 4582 at age 10–12 remained eligible for analysis. To establish a symptom-free baseline population, participants reporting any joint-related symptoms at the respective age were further excluded. This resulted in final analytical samples of 6832 participants at age 5, 3885 at age 8, and 4445 at age 10–12.

**Fig. 2:**
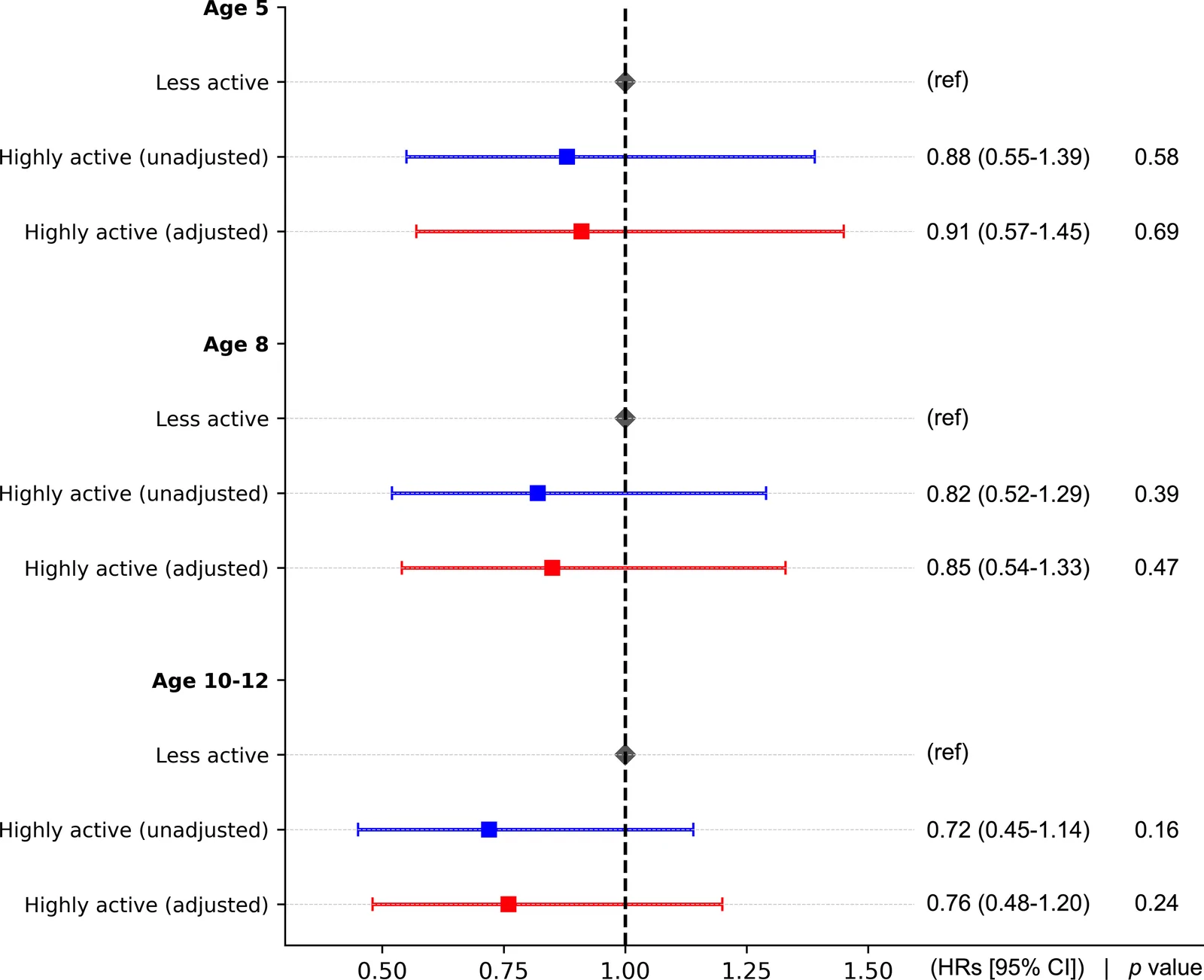
Association between physical activity and risk of JIA. Forest plot showing adjusted hazard ratios and 95% confidence intervals for incident JIA according to physical activity group at age 5, 8, and 10–12 years in symptom free participants. Models adjusted for sex, parental education, and family history of rheumatic disease

In age-specific prospective analyses (Fig. 2), higher PA was consistently associated with lower HRs for JIA across all age groups; however, confidence intervals were wide and included the null value, and no statistically significant associations were observed in either unadjusted or adjusted analyses. Unadjusted HRs (95% CIs) at age 5 was 0.88 (0.55–1.39), at age 8 was 0.82 (0.52–1.29), and at age 10–12 was 0.72 (0.45–1.14). After adjustment for sex, parental education, and family history of rheumatic disease, the association remained similar. Adjusted HRs at age 5 was 0.91(0.57–1.45), at age 8 was 0.85 (0.54–1.33), and at age 10–12 was 0.76 (0.48–1.20). These findings suggest a consistent inverse trend between PA and JIA risk in symptom-free individuals, although the evidence does not support a definitive association.

## Discussion and conclusion

### Principal findings

In this population-based longitudinal cohort study, we examined the association between early-life PA and the risk of JIA using a symptom-free baseline design. Our findings across all age groups suggests that higher PA was associated HRs below unity; however, none of the associations reached statistical significance. These findings indicate a consistent inverse trend between PA and JIA risk, although the results do not support a definitive association.

Importantly, by restricting the analysis to individuals free from both diagnosed JIA and joint-related symptoms at baseline, we ensured that PA preceded any detectable disease process. This design strengthens the temporal interpretation of the results and reduces potential bias related to early disease manifestations.

### Comparison with previous studies

Evidence examining the role of PA in the development of JIA is limited. Most pediatric studies have focused on PA after diagnosis, particularly in relation to disease management and functional outcomes, rather than on disease etiology.

Studies in adult populations have suggested that PA maybe associated with a lower risk of autoimmune disease. For example, a large prospective cohort study by Di Giuseppe et al. [15], reported that higher leisure-time PA was associated with a decreased risk of RA among women. Similarly, Sparks et al. [18], showed that higher PA was associated with lower systemic inflammation and improved immune profiles. However direct comparison with pediatric populations should be interpreted cautiously, as disease mechanisms, exposure timing, and behavioural patterns differ substantially between children and adults.

Our findings are broadly consistent with these studies in showing a protective direction of association, although the lack of statistical significance suggests that the effect of PA on JIA risk, if present, is likely modest. This is not unexpected given the relatively small number of JIA cases which includes strong genetic and immunological components underlying the disease.

### Interpretation and potential mechanisms

PA has been shown to influence immune function, including reductions in systemic inflammation and modulation of immune responses. These mechanisms provide a biologically plausible basis for a potential association between PA and autoimmune disease risk.

However, it is important to consider that JIA is a complex heterogenous autoimmune condition, and PA alone is unlikely to exert a strong causal effect on disease onset. Instead, PA may act as a modifier of underlying risk rather than a primary determinant.

The lack of statistically significant associations in our study is likely attributable to limited statistical power, given the relatively small number of JIA cases within each age-specific analysis. Additionally, the consistency in effect direction across age groups supports the possibility of a modest protective relationship.

### Role of early symptoms and reverse causation

A key methodological challenge in studies of behavioral exposures and disease outcomes is the potential for reverse causation. In the context of JIA, early symptoms such as joint pain, stiffness, or reduced mobility may occur prior to diagnosis and may influence PA levels. We explored this possibility by including individuals with joint symptoms in the control group (see Additional file 1, Table S4, Figure S1, Figure S2). Under these conditions, a statistically significant protective association between PA and JIA were observed at age 10–12 years. However, this association disappeared when individuals with joint symptoms were excluded from the analysis.

This pattern strongly suggests that the observed significance in (see Additional file 1, Figure S1) (without symptom exclusion) may be partly explained by reverse causation, whereby early disease manifestations lead to reduced PA rather than the converse. By restricting our main analysis to a symptom-free population, we minimized this bias and provided a more robust estimate of the association.

### Strengths and limitations

This study has several important strengths. First, it is based on a large population-based longitudinal cohort with repeated assessments of PA across childhood and adolescence. Second, the use of National Health Register data for diagnoses of JIA ensures high validity of outcome ascertainment. Third, the application of a symptom-free baseline design represents a robust approach to addressing reverse causation, which is a common concern in studies of behavioral exposures. Finally, the consistency of findings across multiple age groups enhances the credibility of the observed associations.

Several limitations should be considered. First, the number of JIA cases was were limited, resulting in wide confidence intervals and reduced ability to detect modest associations. JIA is a heterogeneous group of diseases, and associations with PA may differ by subtype. Because of the limited number of cases, we were not able to examine oligoarticular, polyarticular, systemic, enthesitis-related or other JIA categories separately. The results therefore apply to JIA as a combined outcome and may not reflect subtype-specific associations.

Although excluding children with joint-related symptoms reduces the risk that early disease manifestations explain lower activity levels, this approach cannot fully eliminate reverse causation. Subclinical inflammation may precede reported symptoms, and some children may have reduced activity before pain, swelling or limping is recognized by parents or reported in questionnaires.

PA was assessed using self-reported questionnaire data, which may be subject to measurement error and misclassification. Although we adjusted for key confounders, residual confounding cannot be excluded. Additionally, subclinical disease processes may not have been fully captured by the joint symptom variables, although our exclusion criteria likely reduced this bias.

## Supplementary Information

Below is the link to the electronic supplementary material.


Supplementary Material 1


## Data Availability

No datasets were generated or analysed during the current study.

## Abbreviations

JIA: Juvenile idiopathic arthritis
PA: Physical activity
RA: Rheumatoid arthritis
ABIS: All Babies In southeast Sweden
TAS: Total activity score
HRs: Hazard ratios
CI: Confidence intervals
