# Supplementary material for: Early-life physical activity and risk of juvenile idiopathic arthritis: a longitudinal ABIS cohort study using a symptom-free baseline design
Source: Pediatr Rheumatol Online J. 2026 Sep 1;24:57. doi: 10.1186/s12969-026-01266-9 (PMC13536538; doi:10.1186/s12969-026-01266-9)
Supplement: Supplementary file 1 [file 12969_2026_1266_MOESM1_ESM.pdf]

Early-life physical activity and risk of juvenile idiopathic arthritis: A longitudinal ABIS cohort study using a symptom-free baseline design

Table S1: The questionnaire used to measure the Total Activity Score (TAS).

At age 5 years
Hours per day, child watch TV?<br>Hours per day, child play TV/computergame?<br>Hours per day, child spend time in front of a computer?<br>Hours per day, child in motion (playing, jumping)?<br>Hours per week, child at the day care center?
At age 8 years
Hours per day, child watch TV?<br>Hours per day, child spend time in front of a computer?<br>Hours per day, child read books?<br>Hours per week, child organized physical activity?<br>Hours per day, child in motion?<br>How much time/day child spend outside?
At age 10-12 years
Hours per day, do you read books?<br>Hours per day, do you watch TV?<br>Hours per day, do you sit in front of computer?<br>Hours per day, on average is the child in motion?<br>How much the child spends outdoors (per day)?<br>Hours per week, the child has organized physical activity?

Table S2: Threshold comparison at each age point. The threshold ($\mu - 0.5\sigma$) was used in the main analyses. Other thresholds are presented for comparison purposes.

Age	Less Active Group Percentage		
	$\mu - 0.5\sigma$ (%)	$\mu - 0.75\sigma$ (%)	$\mu - 1\sigma$ (%)
Age 5	31.00	17.27	11.86
Age 8	31.00	16.15	9.73
Age 10–12	30.99	20.94	13.91

Table S3: Descriptive characteristics and Sensitivity analysis at each age point. The high Cronbach's alpha values indicate strong internal consistency of the TAS across age groups.

Age	N (Participants)	Mean TAS	SD (σ)	Shapiro-Wilk Stat	Cronbach's Alpha
Age 5	7445	52.06	8.96	0.99	0.98

Age 8	4029	54.12	8.02	0.99	0.98
Age 10–12	4630	55.89	6.85	0.99	0.98

Table S4: Early symptomatic adjustment table for sensitivity analysis.

	Age 5 (%)	Age 8 (%)	Age 10-12 (%)
Total participants	7445	4029	4630
Cases with joint pain	587 (7.9)	112 (2.8)	137 (3.0)
Remaining participants	6858 (92.1)	3917 (97.2)	4493 (97.0)
Early symptomatic cases	14	11	8
Remaining cases with JIA	48	45	32

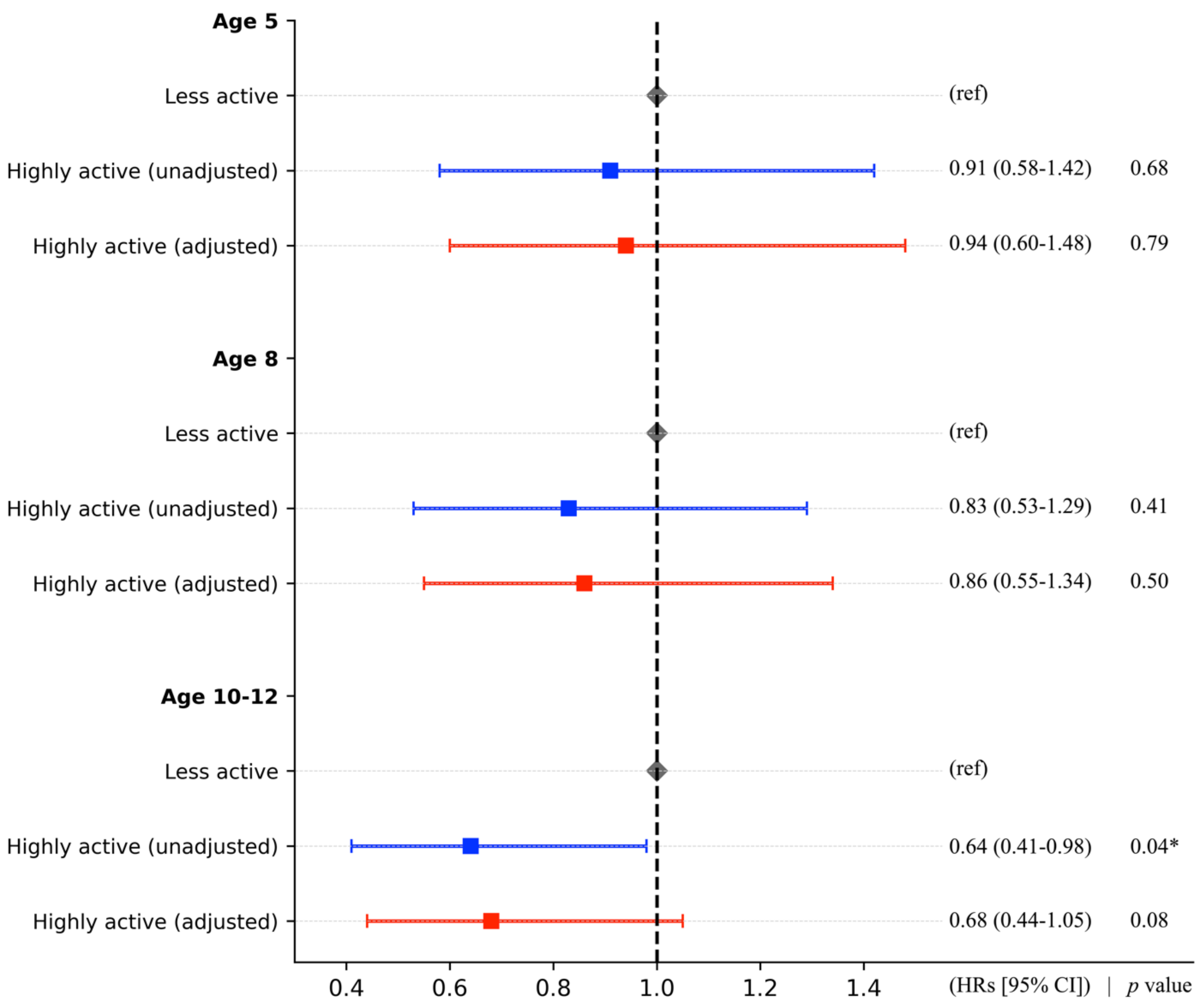

Figure S1: Association between physical activity and risk of JIA without exclusion of joint symptoms.

HRs with 95% confidence interval for the association between physical activity and incident JIA at ages 5, 8, and 10-12 years. In this analysis, participants with prior JIA diagnosis were excluded, however, individuals reporting joint-related symptoms (including pain, swelling, limited mobility, or limping) at the respective age were retained in the study population. Estimates are presented for both unadjusted and adjusted models. Adjusted models include sex, parental education, and family history of rheumatic disease. A statistically significant inverse association was observed at age 10–12; however, this finding should be interpreted with caution.

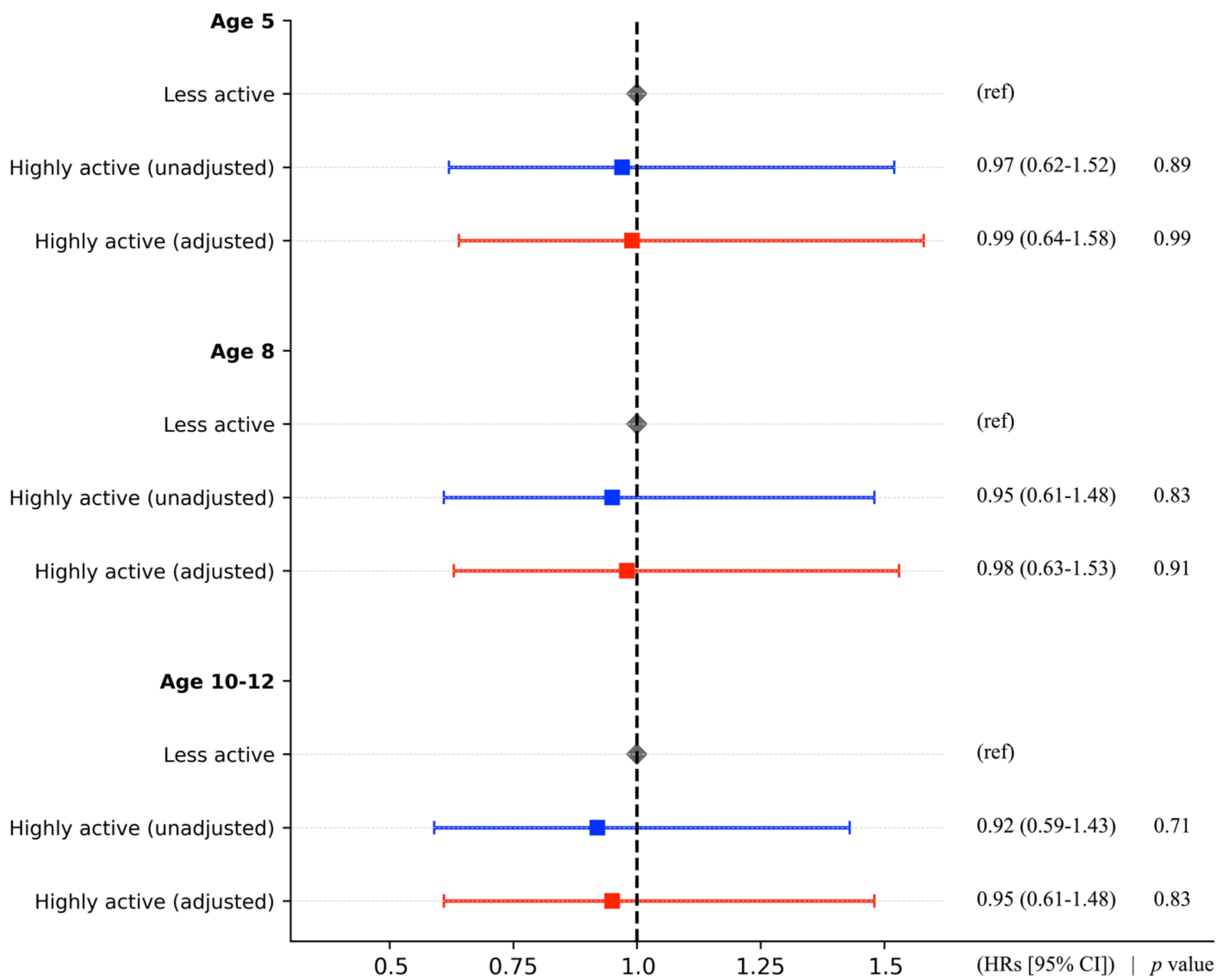

Figure S2: Association between physical activity and risk of JIA after exclusion of participants with joint symptoms (reverse causation analysis).

HRs with 95% confidence interval for the association between physical activity and incident JIA at ages 5, 8, and 10-12 years after exclusion of participants with joint-related symptoms.In this analysis, participants with prior JIA diagnosis and those reporting any joint-related symptoms

(including pain, swelling, limited mobility, or limping) at the respective age were excluded to minimize potential reverse causation. Estimates are shown for both unadjusted and adjusted models. Adjusted models include sex, parental education, and family history of rheumatic disease. Compared with the analysis including symptomatic individuals (Figure S1), the previously observed statistically significant association at age 10-12 was attenuated and no longer statistically significant, suggesting that early joint symptoms may have influenced physical activity levels prior to diagnosis.
